# Chemotherapy-Related Toxicity, Nutritional Status and Quality of Life in Precachectic Oncologic Patients with, or without, High Protein Nutritional Support. A Prospective, Randomized Study

**DOI:** 10.3390/nu9101108

**Published:** 2017-10-11

**Authors:** Monika Ziętarska, Joanna Krawczyk-Lipiec, Leszek Kraj, Renata Zaucha, Sylwia Małgorzewicz

**Affiliations:** 1 Department of Clinical Nutrition, Medical University of Gdańsk, 80-211 Gdańsk, Dębinki 7, Poland; monikatkacz@gumed.edu.pl; 2 Department of Haematology, Oncology and Internal Medicine, Medical University of Warsaw, Banacha 1a, 02-097 Warszawa, Poland; leszekkraj@gmail.com; 3 Department of Oncology and Radiotherapy, Medical University of Gdańsk, 80-211 Gdańsk, Dębinki 7, Poland; renata.zaucha@gmail.com

**Keywords:** colorectal cancer, chemotherapy, nutritional status, appetite, quality of life, oral nutritional supplementation

## Abstract

Background: Cancer disease is usually associated with impaired nutritional status, which is one of the factors contributing to deterioration of the results of surgery, chemotherapy or radiotherapy. Objectives: The aim of the study was to determine whether nutritional support with high protein (ONS) in adult oncologic patients in the first step of cancer cachexia—asymptomatic precachexia, has an influence on the toxicity of systemic therapy. However, secondary endpoints were established: to determine whether high protein ONS influences the nutritional status, the quality of life, and the performance status. Materials and Methods: A total of 114 persons aged 40–84 years old with colorectal cancer were examined. Based on the randomization, 47 patients were qualified to the interventional group (ONS group) and 48 to Control group. To evaluate the nutritional status NRS-2002 (Nutritional Risk Screening), SGA (Subjective Global Assessment), SCRINIO (SCReenIng the Nutritional status In Oncology) Working Group classification, VAS (Visual Analog Scale) for appetite was used. FAACT (Functional Assessment of Anorexia/Cachexia Therapy) questionnaire was used for assessment of the quality of life. The health status of patients was evaluated based on the Karnofsky Performance Scale. Anthropometric measurements were done. Results: Severe complications of chemotherapy, which caused the end of treatment, a slight complication of the gastrointestinal tract such as diarrhea grade 2 according to ECOG (Eastern Cooperative Oncology Group) score regardless of the studied group, were observed. There were no statistical differences in the number and severity of the observed complications, i.e., neutropenia, leucopenia, thrombocytopenia, anemia, abdominal pain, nausea and vomiting, and diarrhea. During the follow-up the significant changes of SGA, VAS, albumin and prealbumin were observed between groups. In the ONS group an improvement in nutritional status was noticed (increased appetite VAS, *p* = 0.05; increased points in SGA, *p* = 0.015, and increased levels of albumin and prealbumin, *p* = 0.05). In Control group nutritional status was stable during observation. The performance status and quality of life were stable in both groups. No statistical differences between groups (ONS vs. Control) in the numbers for disqualification, resignation, delay in treatment, or dose reduction were observed. Conclusions: Results of the study did not indicate that nutritional support in precachectic oncologic patients influenced the toxicity of systemic therapy. High protein nutritional support improved nutritional status assessed by SGA, VAS for appetite, albumin, and prealbumin. The performance status and quality of life were stable throughout the observation and were not changed under the supplementation.

## 1. Background

Cancer is a systemic disease, since even in the early stages malignancies are accompanied by homeostatic imbalance, including metabolic deregulation and increased catabolism. These abnormalities may initially seem poorly discernible in the clinic, but with disease progression they may aggravate; and may cause overt cancer-related cachexia. 

It is known that cachexia is an independent negative prognostic factor, a leading cause of impairment of the quality of life, and that it produces functional deterioration of oncologic patients. Various frequencies of cachexia in different malignancies are also well described, and may result from their different biology or, presumably, from unknown individual factors [1,2,3,4,5,6,7,8,9]. Finally, it should be noted that in recent years the concept of cancer cachexia was frequently misregarded as simple undernutrition, resulting in the inefficacy of the undertaken interventions.

However, according to the international consensus, cancer-related cachexia is a complex multifactorial syndrome defined by an ongoing loss of lean body mass (mainly skeletal muscle mass) that cannot be fully reversed by conventional nutritional support as a single intervention, and leads to a progressive functional impairment of the whole organism [2]. 

The pathogenesis of cachexia is not completely elucidated, yet negative protein and energy balances are its crucial elements: these being the consequence of deregulation of multiple intermediate metabolic pathways, latent inflammatory state and anorexigenic action of multiple biologically-active factors [1,2]. 

Moreover, it should be stressed that clinical pictures of metabolic disorders in cancer form a continuum; beginning from precachexia (stage I), through cachexia (stage II) and ending with refractory cachexia (stage III) [1,10] or according to SCRINIO (SCReenIng the Nutritional status In Oncology) Working Group classification—4 classes—from asymptomatic precachexia (class 1) to symptomatic cachexia (class 4) [11]. 

The cancer cachexia could be associated with increased toxicity of cytotoxic treatment, and frequently necessitate premature termination of therapy. Furthermore, cachectic patients have poor performance status. So, a vicious cycle develops: cachexia reduces the possibility of use of cytotoxic agents, thus leading to disease progression, and this in turn promotes the development of resistant cachexia. The result is not only an unfavorable prognosis, but also poor quality of life for patients. Unfortunately, a sole nutritional intervention is inefficient in these cases, and its support with targeted pharmacotherapy requires further research. Thus, it is important to undertake actions aimed at prevention of developed cachexia (stage II and III) or postponing its occurrence [1,2,12,13].

Cancer-related precachexia—the first step of cancer-related cachexia—requires special attention. The optimal nutritional management of patients with precachexia could contribute to improvement of tolerability of oncologic therapy; as well as administration of due dose density, translating into higher treatment efficacy and improvement or preservation of the current nutritional and metabolic status. A positive impact on the quality of life and performance status is also proposed [7,8,10,13]. 

The patients with precachexia may have particular benefits from the implementation of nutritional support. Nevertheless, such data is lacking in the literature. Of course, maintaining or improving nutritional status in patients undergoing chemotherapy or chemo-radiotherapy is extremely important, as it is associated with improved prognosis. And indeed, this is the primary endpoint of many clinical trials. However, it seems that both the nutritional status and the toxicity of chemotherapy are closely related. 

However, the optimal nutritional management of precachectic oncologic patients, remains unclear. Presently, no nutritional support leading to an increase of nutrient delivery is routine use in asymptomatic precachectic patients. Furthermore, there are no specific guidelines on the requirements for nutritional support in these persons. Oral nutritional supplements (ONS), however, may be of some importance. 

Unfortunately, currently there are no standards for nutritional management in precachectic stage., It is especially unclear whether nutritional support should be implemented in preemptive strategy in the first step of cancer cachexia—asymptomatic precachexia. 

Furthermore, an unresolved question concerns the clinical justification of ONS use, which could also have economical consequences. The discussed products allow for delivery of nutritional elements in amounts covering the demand, in small volumes and in a form convenient for consumption. It should be noted that such a supply is often difficult to assure with nutrition using natural products only. Nevertheless, lack of data regarding the tolerability of ONS/intake ONS compliance remains another unsolved problem. Many products are characterized by high nutritive density and high osmolarity, which are associated with a potential risk of the induction of gastrointestinal disorders or paradoxical anorexia. 

Elucidation of the discussed issues seems especially important in view of possible improvements to tolerance of chemotherapy, quality of life, nutritional status and prognosis in cancer patients; as well as potential savings of healthcare funds.

### The Aims of the Study

The aim of the study was to determine whether nutritional support with high protein ONS in adult oncologic patients in the first step of cancer cachexia—asymptomatic precachexia, influences the toxicity of systemic therapy.

However, secondary endpoints were established: to determine whether high protein ONS influences the nutritional status, the quality of life and the performance status.

## 2. Material and Methods (and Patients)

The study received approval of the Ethics Committee of the Medical University of Gdansk (no. NKBBN/412/2014). Informed consent was obtained from all individual participants included in the study. The study was conducted in the Department of Oncology and Radiotherapy in the Medical University of Gdańsk. Patients were treated by oncologists, nutritional assessment was performed by an internal medicine physician and clinical dietitian.

**Figure 1:**
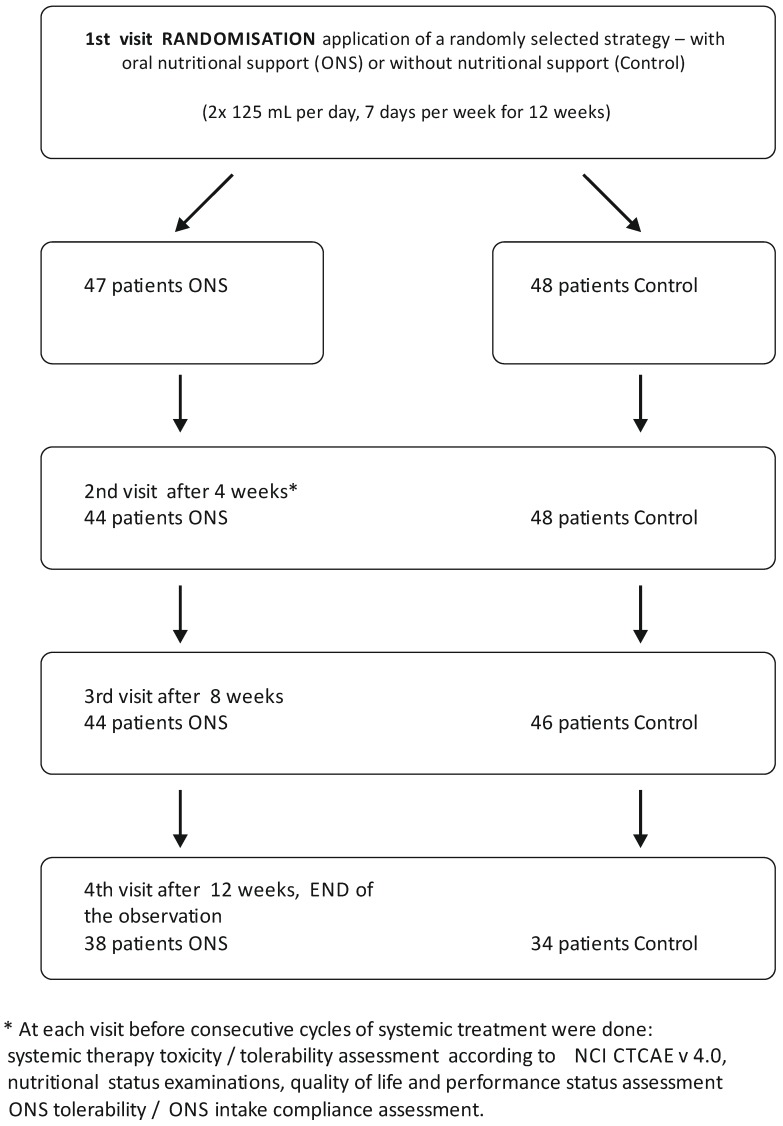
The scheme of the study.

A total of 114 persons aged 40–84 years old were examined. Based on the randomization (mode of randomization—computer-aided allocation to study groups) 47 patients were qualified to the interventional group (ONS group) and 48 to Control group (scheme of the study Figure 1). The characteristics of the study groups are presented in Table 1.

The results in the studied groups were collected over the course of 12 weeks of observation, with visits every 4 weeks. During each visit, assessment of the nutritional status, performance status based on the Karnofsky scale and quality of life, compliance with recommendation and tolerability of ONS treatment were done.

For each visit in the ONS group for the assessment of patients’ compliance, we provided patients with a fixed number of supplement packets, and instructed them to return the unconsumed packets at the next visit. Moreover, on the sheet, the patient recorded the number of drinks consumed each day. 

### 2.1. Inclusion Criteria

- Histological confirmed diagnosis of colorectal cancer (CRC) in clinical stage II-IV according to TNM UICC 2010.
- Successful qualification to first line chemotherapy according to protocols with: 5-fluorouracil, leucovorin, oxaliplatin (FOLFOX-4) or 5-fluorouracil, leucovorin, irinotecan (FOLFIRI); 100% dose of chemotherapy (no dose reduction).
- Performance status at least 80% according to Karnofsky scale.
- Cancer-related asymptomatic precachexia diagnosed according to SCRINIO Working Group.
- Absence of contraindications to oral nutrition and practicable realization of oral nutrition.
- Absence of severe, decompensated concomitant diseases—e.g., diabetes, hepatic insufficiency, renal insufficiency (K/DOQI stage ≥ 2).
- Signed informed consent for participation in the study.

### 2.2. Exclusion Criteria

- Diagnosis of a malignant neoplasm in clinical stage I according to TNM UICC 2010.
- Disqualification from oncologic treatment.
- Cancer cachexia or cancer anorexia–cachexia syndrome.
- Poor performance status—Karnofsky scale < 80% or WHO/ECOG (World Health Organisation/Eastern Cooperative Oncology Group) 2–4.
- Contraindications to oral nutrition or to high protein nutrition (e.g., hepatic or renal failure).
- Regular nutritional support at the moment of qualification to the study.
- Patient incompliance at the moment of qualification to the study.

All patients had histopathologically confirmed diagnosis of colorectal cancer. On the basis of the TNM classification 30 persons had the fourth stage (31.6%), 50 persons (52.7%)—third stage, and 15 persons (15.8%)—the second stage. Metastases to lymph nodes were diagnosed in 28 persons. Distant metastases: lung, ovary, liver, bladder, kidney, adrenal glands were found in 21 persons. 

71 patients (74.7%) were in the second clinical stage (G2), 20 patients (21.1%)—in the third stage (G3), and 4 patients (4.2%)—in the fourth stage (G4). The location of the tumor was as follows:

- caecum—4.0%
- ascending colon—58.6%
- sigmoid colon—16.0%
- rectum—21.3%.

Patients were qualified to receive the treatment with chemotherapy. A total of 41.0% patients in the study were qualified to receive FOLFOX regimen and the rest of the patients were qualified to the following regimens FOLFIRI, LF, CLF, XELOX.

### 2.3. Dropout Details

The full 12 weeks follow-up period was completed by 72 patients. The shorter observation period was associated with resignation from the study, early termination of chemotherapy because of severe complications, transfer to another center. 

In the ONS group, 6 patients resigned from the study (gave up on taking supplements). 

In Control group, 2 patients moved to another oncology center, 1 patient resigned from the oncology treatment, 5 patients resigned from the study (no time for additional visits, fatigue). The above data are presented in Appendix: Table A1.

There were no statistical differences between groups (ONS vs. Control) in the number of resignation from the nutritional treatment.

Chemotherapy-related toxicity was graded according to National Cancer Institute Common Terminology Criteria for Adverse Events (NCI CTCAE Version 4.0).

Every 4 weeks, on every visit, data on toxicity of chemotherapy such as neutropenia, leukopenia, anemia, thrombocytopenia, diarrhea, vomiting, abdominal pain, and sepsis or other symptoms were noted.

In addition, delayed chemotherapy and dose reduction due to toxicity were recorded.

### 2.4. Nutritional Status, Performance Status and Quality of Life

The following methods for determination of the nutritional status were used in the study:

Body mass measurement with the use of the scale (Tanita BC 420).

Body mass index (BMI) calculation on the basis of the following formula:

BMI = body weight/height^2^ (kg/m^2^)

BMI classification was adopted:

<18.5—underweight
18.5–24.9—normal body weight
25.0–29.9—overweight
<30.0—obesity

The precachexia was diagnosed on the basis of SCRINIO Working Group Classification i.e.,

- unintentional weight loss in the past six months <10%
- no anorexia.

The cachexia was diagnosed when the weight loss was >10% in the past six months.

Also, the following scores were used:

Nutritional Risk Screening (NRS-2002)—method is based on the evaluation of unintentional weight loss, BMI, consumption quantity, clinical condition and age. Gaining three or more points means the risk of malnutrition.

Subjective Global Assessment (SGA)—on the basis of this method consisting of an interview, unintentional body weight loss, clinical status and physical examination patients are qualified to the following groups: well-nourished (7 or 6 points), moderately malnourished (5, 4, 3 points) and severely malnourished (1 or 2 points).

Visual Analog Scale for Appetite (VAS) was completed by patients on the basis of a patient’s feelings regarding appetite during the previous seven days. VAS for appetite is a 100-mm line where 0 mm means “I had no appetite at all”, and 100 mm means “My appetite was very good”. VAS score is obtained by measuring the distance (in millimetres) from 0 point “I had no appetite at all” to the point selected by a patient. The lower the score—the worse appetite. It was assumed that below 70 mm appetite is moderately reduced, and below 50 mm—severely reduced (weak).

Functional Assessment of Anorexia/Cachexia Therapy (FAACT) is a measure of the quality of life, and it is completed by the patient, it has a total of 42 questions. Patients mark 0 (none)—4 (very much) for each question. This tool consists of five subscales: functional well-being (7 items), physical well-being (7 items), social/family well-being (7 items), emotional well-being (6 items) and other aspects, including appetite (13 items). The maximum score is 168. The lower the score, the lower the overall quality of life.

Karnofsky scale allows one to quantify the overall state, and quality of life, of a patient with neoplastic disease who qualifies to receive chemotherapy. The score has a range from 0 to 100, where 100 represents the ideal state and 0 represents death.

### 2.5. Laboratory Measurements

At each visit, all participants were measured in serum concentration of albumin, prealbumin, triglycerides, total cholesterol, C-reactive protein (CRP), ferritin, complete blood count (WBC—white blood cells, PLT—platelets, NEUT—neutrocytes, Hb—hemoglobin).

### 2.6. Nutritional Intervention

Patients were treated by high-energy, high-protein, oral liquid nutritional supplements; dosage: A total of 2 × 125 mL per day, 7 days per week for 12 weeks (3 months). One drink contained 300 kcal, 18 g of protein. It contained carbohydrates, lipids, minerals, trace elements and vitamins. It is clinically free from lactose and gluten-free. Patients were given a choice of 4 flavours and could change them during supplementation.

### 2.7. Statistical Analysis

Results are expressed as percentages (for categorical variables), mean and standard deviation. The assumption of normality was verified with the Kolmogorov-Smirnov test. A *p*-value < 0.05 was considered to be statistically significant. To assess correlations among the evaluated variables, Pearson’s correlation coefficient (r) was used. A multifactorial regression method was used to establish relationships between variables (between albumin, nutritional parameters and inflammation marker—CRP). 

Comparisons between two groups were assessed with a Student’s *t*-test or a Mann–Whitney test, as appropriate. Compared also were the incidence of events (e.g., dose reductions or delay of chemotherapy cycle) in study groups. Basic inference was based on the Fisher–Boschloo double-sided test, Chi-square test (with Yates’ correction). The change in value between the first and fourth visits (the value for the fourth visit minus the value for the first visit) was compared between groups. The comparison was made using the two-sided Welch test (a generalized *t*-Student test, accounting for the possibly different variances in the groups. Whenever we studied if the change in a variable differs between the two subgroups, we compared the mean of the differences between the first and the fourth visit.

Statistical processing of the results was performed with the use of the statistical software STATISTICA PL v 12.0 (Statsoft, Kraków, Poland).

## 3. Results

### 3.1. Baseline Results

In the whole study group the mean unintentional body weight loss during the previous six months was 6.7 kg (median 6 kg) and ranged from 1.1 kg to 15.0 kg. The mean BMI was within normal limits and was 24.5. In NRS-2002 assessment: 76% in the study group received three or more points—which indicates the risk of malnutrition, or malnutrition, and indicates the need for nutritional intervention. Similarly to NRS-2002—in SGA 73.3% of patients were classified as presenting mild/moderate malnutrition. 37.0% of patients had moderate decreased appetite and 6.0% had poor appetite. Results of the Karnofsky scale indicated the state of normal activity, minor signs and symptoms of the disease among the majority of the patients. FAACT—average score (median 78.5 points) indicated a reduction in the quality of life of the patients in all aspects of functioning.

At the baseline patients in the ONS group had higher albumin (*p* = 0.04), prealbumin (*p* = 0.02) and lower body mass and BMI (*p* = 0.04) (Table 2).

### 3.2. Follow—Up Period

The comparisons between the 1st and 4th visits in the study groups are presented in the Table 2. 

During the follow-up the significant changes of SGA, VAS, albumin and prealbumin were observed between groups. In ONS group improvement in nutritional status was observed (increased appetite VAS, *p* = 0.05; increased points in SGA, *p* = 0.015, and increased levels of albumin and prealbumin, *p* = 0.05). 

In Control group nutritional status was stable during observation.

**Figure 2:**
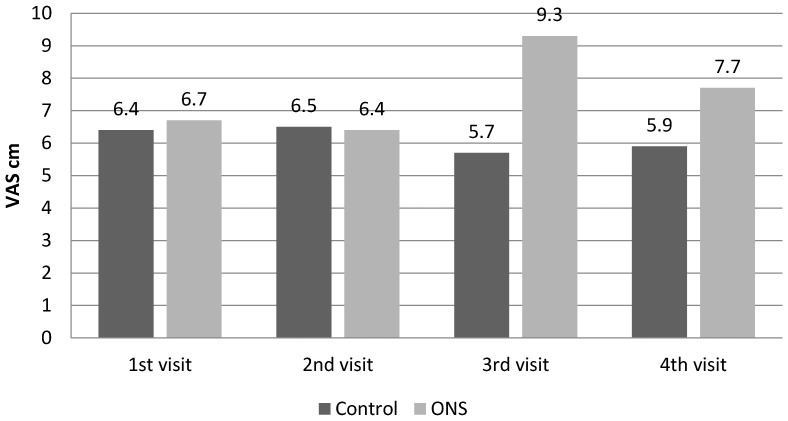
The changes of appetite (visual analog scale for appetite -VAS) during observation in both groups.

The significant differences between the ONS and Control groups were found after 4 weeks in the SGA (6.8 vs. 5.1 points; *p* = 0.004); the appetite significantly increased in the ONS vs. Control group after 8 weeks of observation (9.3 vs. 5.7 cm; *p* = 0.001) and also after 12 weeks (7.7 vs. 5.9 cm; *p* = 0.001) (see Figure 2).

During the 3rd visit there was a difference in albumin concentration (ONS vs. Control 39.9 vs. 35.5; *p* = 0.01, this change was maintained during the 4th visit (at the limit of statistical significance).

Prealbumin was statistically significantly higher in the ONS group in comparison to Control during the 4 visit (35.0 vs. 29.1, *p* = 0.001). Concentration of prealbumin was correlated positively with albumin level (r = 0.45, *p* = 0.03) and negatively with CRP (r = −0.35, *p* = 0.03).

**Figure 3:**
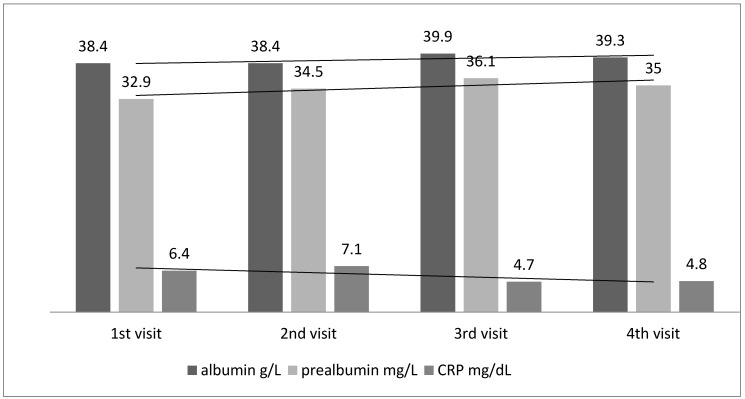
The changes of albumin, prealbumin and C-reactive protein (CRP) level during observation in ONS group (1—1st visit, 2—2nd, 3—3rd and 4th visit).

Multiple regression analysis showed that albumin concentration in the ONS group was associated with CRP level (Table 3). However, the changes in albumin, prealbumin and CRP levels during the study in the ONS and Control groups are presented in Figure 3.

#### 3.2.1. Chemotherapy-Related Toxicity 

Severe complications of chemotherapy, which caused the end of treatment a slight complication of the gastrointestinal tract such as diarrhea grade 2 according to ECOG score regardless of the studied group were observed. The causes of early termination of chemotherapy due to severe complications are presented together with dropout analysis in Appendix: Table A1.

During 12-months of observation in ONS group 1 patient was hospitalized for sepsis and 3 patients were disqualified from chemotherapy because of progression of disease and worsening of overall condition. 

In Control group 1 patient had sepsis, 5 patients were disqualified from chemotherapy because of progression of disease, worsening of overall condition, allergic reactions to treatment.

During follow-up—delayed administration of the next chemotherapy cycle, or half-dose reduction due to thrombocytopenia, leucopenia or neutropenia were reported in 3 patients in the ONS group and 7 in the Control group. On the other hand, the delay of chemotherapy for an average of 7 days was in 14 subjects (total 18 cycles) in the ONS group and 10 subjects (13 cycles) in the Control group.

There were no statistical differences between groups (ONS vs. Control):

- in the number and severity of the observed complications, ie neutropenia, leucopenia, thrombocytopenia, anemia, abdominal pain, nausea and vomiting, and diarrhea
- in the number of dose reductions (*p* = 0.30), delay of chemotherapy cycle (*p* = 0.24) or disqualification from continuation of chemotherapy.

#### 3.2.2. Compliance/Tolerability

In ONS group compliance was high—80% of patients made recommendations in excess of 75%. 

Also, tolerability of the ONS treatment was good. There were no differences between the groups (ONS vs. Control) in number and severity of nausea, vomiting, diarrhea, abdominal pain.

## 4. Discussion

### 4.1. Nutritional Status

An extremely important clinical issue in oncology is the problem of malnutrition or high risk of deterioration of nutritional status, lack of appetite and subclinical inflammation.

High percentages of patients with significant weight loss have already been diagnosed early as onset of cancer—depending on the location of the cancer, and the stage of the disease, it ranges from 40 to over 80% [14,15,16,17]. The percentage of patients who are diagnosed with anorexia—often in the form of a malignant anorexia-cachexia syndrome—and subclinical inflammatory disease is also not less.

Both subclinical inflammation, as well as metabolic disorders and abnormal nutritional status as a consequence, are highly unfavorable prognostic factors. In addition, the negative impact of the above on quality of life and functional independence of patients at various stages of cancer have also been found.

It is known that nutritional interventions in patients with malnutrition is unequivocally indicated, although it may not be fully effective due to co-existing metabolic abnormalities with increased catabolic processes. It would be prudent to undertake earlier nutritional interventions, for example, already at the stage of precachexia.

This study included the population of patients who met the criteria for diagnosis of cancer precachexia. They were patients with colorectal cancer in stage II–IV according to the TNM classification.

In the study group, despite the normal or elevated BMI, it was found that the majority of patients were at high risk of malnutrition, or mild malnutrition criteria were met. The assessment of nutritional status was done comprehensively. Recommended scales for assessment of nutritional status and biochemical indicators as mentioned above. It is worth stressing that such a comprehensive assessment is necessary to obtain adequate results, since the measurement of body mass and BMI itself is insufficient and not entirely reliable.

At the start of the study, appetite in most patients was moderate (median VAS was 6.5 mm). However, it should be noted that the study program excluded patients with fully symptomatic anorexia. Despite the differences in BMI and body mass between study groups, at the beginning of the study unintentional body mass lost in both groups were similar and less than 10%.

Unfortunately, in the ONS group albumin and prealbumin concentration were significantly higher in comparison to the Control group at the beginning of the study, but statistical analysis after 3-months of observation considered the degree of changes during follow-up. But we can not exclude that in the case of patients with lower albumin and prealbumin a positive effect of supplementation would be even more noticeable. Statistically significant improvement in nutritional status was observed on the third visit in ONS group; while in the Control group, at the same time, a decrease of albumin and prealbumin levels was observed. Moreover, after completion of follow-up in the ONS group, compared to the Control group, patients presented statistically better appetite and higher albumin and prealbumin levels.

Thus, the implementation of oral high protein diet support already at the stage of precachexia appears to be significant and clinically relevant. In this study, it was found to improve the nutritional status of patients receiving ONS. In addition, the use of a high protein supplement did not negatively affect appetite. On the contrary, patients from the ONS group presented an improvement in appetite as compared to the Control group.

It is also worth emphasizing the significantly higher serum albumin and prealbumin levels in patients receiving ONS, which may have a long-term beneficial prognostic significance. The literature highlights the significant role of these nutritional markers in patients with cancer of various organs, including those with colorectal cancer [13,18,19].

Higher serum albumin levels are associated with improved prognosis, fewer complications after oncological treatment, and longer survival time. In a large retrospective analysis of more than 40,000 patients with colorectal cancer, hypoalbuminemia (<3.5 g/dL) was a strong correlator with longer hospitalization time and greater complications after surgical management of colorectal cancer [18]. In another study, baseline hypoalbuminemia (<3.5 g/dL) was found to be an independent factor associated with shorter survival time after primary tumor resection [19]. Similarly, in patients with relapsed or unresectable colorectal tumors, low albumin concentrations correlated with shorter total survival (OS) [20].

Probably, in our study the too short observation time made it impossible to find a similar relationship—better prognosis in better nourished patients.

### 4.2. Inflammation

As mentioned above, subclinical inflammation, which occurs in a significant proportion of patients with solid tumors, contributes to many metabolic disorders (e.g., insulin resistance), anorexia and, consequently, to abnormal nutritional status.

Indeed, the investigated inflammatory marker—C-reactive protein (CRP) indicated the presence in most of the subjects of chronic inflammation, although no clinical evidence of infection was observed. Moreover, what was extremely important was the negative correlation between CRP and biochemical parameters of nutritional status (albumin, prealbumin) and appetite assessed by VAS. It is also worth noting that after 2 months of observation CRP levels have decreased in the ONS group, and regression indicates that CRP was a statistically significant factor affecting albumin concentration.

The above observation also seems to be very clinically relevant. It appears that the implementation of high-protein oral nutritional support in patients with precachexia reduces the expectations of subclinical inflammation while contributing to improved nutritional status.

### 4.3. Chemotherapy-Related Toxicity 

In both study groups, patients with mild to moderate complications (systemic adverse events) were the most commonly reported. Diarrhea, as well as nausea and lack of appetite, were observed in grade 1–2 (acc. NCI CTCAE) several days after receiving the drug. These are the most frequently mentioned in the literature, the adverse effects of chemotherapy primarily due to the gastrointestinal toxicity of cytostatics.

Laboratory results (e.g., peripheral blood morphology) were stable during follow-up—delayed administration of the next chemotherapy cycle, or half-dose reduction due to thrombocytopenia, leucopenia or neutropenia were similar in both groups.There were no direct statistically significant differences in chemotherapy related toxicity between the two study groups. However, it seems highly likely that such a difference would occur in the case of longer follow-up times, and more chemotherapy cycles. This indicates an improvement in nutritional status and a reduction in inflammatory parameters in patients receiving nutritional support.

### 4.4. Limitations and Advantages of the Study

Our study is one of the first studies of early nutritional support during chemotherapy, and the first in CRC. The monthly visits with extensive data collection in both groups, including a strong focus on nutrition status, helped minimize placebo effect. The high compliance is also a strength of the study. The limitation of this study is primarily the short observation period, as well as patient resignation during the study. Patients most often reported lack of time, fatigue, malaise, and in ONS group, difficulty maintaining the recommended dose of the supplement, bad taste of the supplement. However, it is worth emphasizing the good tolerance of dietary treatment, the absence of differences in gastrointestinal symptoms between groups, and the high level of compliance of patients with recommendations for nutritional treatment.

It appears that the implementation of high-protein oral nutritional support at an early stage of metabolic disorders and nutritional status (i.e., precachexia) is a good method to reduce the risk of deepening malnutrition. It is also worth stressing that such action reduces the exponents of subclinical inflammation, which is the main pathophysiological cause of the development of malignant anorexia-cachexia syndrome. Taking into account the results of the study, the main endpoint should be the nutritional status rather than toxicity. Determining the effect of supplementation on chemo-toxicity has resulted in much longer follow-up and more patients.

Further studies, and longer follow-up time, are advised to answer the question—does the use of high-protein dietary support at the stage of precachexia involve a prolongation of overall survival in patients with colorectal cancer? We are still looking for new therapies that will help to improve the prognosis of patients with cancer. It is also worth to pay attention to nutritional aspects, and their possible role in the prognosis.

## 5. Conclusions

1. Results of the study did not indicate that nutritional support with high protein ONS in adult oncologic patients in the first step of cancer cachexia—asymptomatic precachexia, had an influence on the toxicity of systemic therapy.
2. High protein dietary support improves nutritional status in colorectal cancer patients with precachexia.
3. The performance status (based on Karnofsky scale) and quality of life were stable throughout the observation, and was not changed under the supplementation.
4. Tolerability of the ONS treatment was good. There were no differences between the groups (ONS vs. Control) in number and severity of nausea, vomiting, diarrhea, abdominal pain.

## Figures and Tables

**Table 1:** The characteristics of the study groups.

Parameters	ONS *n* = 47	Control *n* = 48
Age years mean ± SD	65.00 ± 9.97	63.66 ± 9.39
Females/Males *n*	20/27	26/22
Stadium G *n*/%	G2 = 33 (70.2%)	G2 = 38 (81.2%)
	G3 = 11 (23.4%)	G3 = 9 (18.7%)
	G4 = 3 (6.4%)	G4 = 1 (2.1%)
Metastases *n*/%	*N* = 35 (74.5%)	*n* = 40 (83.3%)
Stoma *n*/%	*N* = 23 (48.9%)	*N* = 13 (27.0)
Chemotherapy type *n*/%	FOLFOX *n* = 16 (34.1%)	FOLFOX *n* = 17 (35.4%)
	FOLFIRI *n* = 7 (14.8%)	FOLFIRI *n* = 8 (16.6%)
	Other *n* = 24 (51.1%)	Other *n* = 23 (47.9%)

**Table 2:** The comparison between 1th and 4th visit in the study groups: ONS and Control.

Parameters	ONS *n* = 47	Control *n* = 48	ONS *n* = 38	Control *n* = 34	*p* ONS vs. Control *
1st visit			4th visit		
Nutritional Status					
BMI kg/m^2^	23.99 ± 3.26	26.5 ± 4.65	24.0 ± 5.2	25.32 ± 8.47	0.44
SGA points	4.68 ± 0.91	5.10 ± 0.55	5.4 ± 0.55	5.2 ± 0.59	0.05
NRS-2002 points	3.19 ± 0.77	3.02 ± 0.56	2.5 ± 0.6	2.6 ± 5.00	0.47
VAS cm	6.44 ± 2.68	6.40 ± 2.00	7.79 ± 1.7	5.90 ± 2.22	0.0001
Albumin g/L	37.89 ± 4.65	35.68 ± 5.53	39.15 ± 4.28	35.9 ± 5.30	0.006
Prealbumin mg/L	31.83 ± 7.03	28.13 ± 8.60	34.68 ± 4.28	29.14 ± 7.76	0.001
Biochemistry					
WBC ×10^9^/L	6.66 ± 2.15	6.71 ± 2.96	5.35 ± 1.8	6.62 ± 3.60	0.36
NEUT ×10^9^/L	3.74 ± 1.69	3.78 ± 2.18	2.6 ± 1.5	3.59 ± 3.46	0.12
RBC ×10^9^/L	4.15 ± 0.52	4.27 ± 0.68	4.04 ± 0.5	3.96 ± 0.59	0.49
Hb g/dL	11.61 ± 1.53	12.09 ± 2.06	11.7 ± 1.4	11.8 ± 1.90	0.91
Plt ×10^9^/L	279.36 ± 121.37	263.37 ± 109.07	199.5 ± 65.9	200.7 ± 82.20	0.94
CRP mg/dL	7.14 ± 4.33	8.76 ± 12.21	5.0 ± 2.9	6.1 ± 3.54	0.13
Ferritin µg/dL	52.89 ± 44.05	104.54 ± 107.39	42.5 ± 33.9	92.9 ± 88.22	0.001
Total Cholesterol mg/dL	176.20 ± 48.02	196.53 ± 97.64	173.5 ± 46.0	172.6 ± 40.1	0.93
Triglycerides mg/dL	149.88 ± 72.96	159.07 ± 53.76	176.18 ± 99.7	148.9 ± 41.4	0.14
Quality of Life					
FAACT points	78.27 ± 13.28	75.89 ± 13.82	76.8 ± 13.2	74.96 ± 12.8	0.55
Performance Status					
Karnofsky scale %	91.48 ± 7.21	93.95 ± 6.43	93.24 ± 6.86	92.00 ± 6.64	0.45

The data are presented as means ± SD. BMI body mass index; SGA—subjective global assessment; NRS-2002 nutritional risk screening; VAS—visual analog scale for appetite; WBC—white blood cells; NEUT—neutrocytes; RBC—red blood cells; Hb—hemoglobin; Plt—platelets; CRP—C reactive protein; FAACT—Functional Assessment of Anorexia/Cachexia Therapy. * The change in value between the first and fourth visits for patients who completed full observation (the value for the fourth visit minus the value for the first visit) was compared between groups (Welch test).

**Table 3:** The multivariate regression. The dependent variable: albumin.

The Dependent Variable	B	Standard Error	Beta	*p*
Constant			52.00583	0.000013
SGA	−0.005915	0.196710	−0.04437	0.976225
NRS-2002	−0.205971	0.175000	−1.32157	0.249113
VAS	−0.042075	0.200423	−0.10725	0.835241
CRP	−0.524951	0.196874	−0.78814	0.012590
Returned drinks	0.000193	0.172006	0.00006	0.999115

SGA—subjective global assessment; NRS-2002 nutritional risk Screening; VAS—visual analog scale for appetite; CRP—C reactive protein.

## Appendix A

**Table A1:** The reasons for dropout of the study and causes of early termination of chemotherapy due to severe complications.

1st Visit	Randomization Start Observation and Intervention	
	Control Group *n* = 48	ONS Group *n* = 47
2nd visit	*n* = 48	*n* = 44 *2 patients resigned from the study (gave up on taking supplements), 1 patient was disqualified from chemotherapy (kidney injury)
3rd visit	*n* = 46 ** 2 patients moved to another oncology center	*n* = 44
4th visit	*n* = 34 *** 1 patient was hospitalized for sepsis 5 patients were disqualified from chemotherapy because of progression of disease, worsening of overall condition, allergic reactions to treatment (oxaliplatin), 1 patient resigned from the oncology treatment, 5 patients resigned from the study (no time for additional visits, fatigue)	*n* = 38 *** 1 patient was hospitalized for sepsis 1 patients was disqualified from chemotherapy because of progression of disease and worsening of overall condition, 4 patients resigned from the study (gave up on taking supplements)

* Incidents between 1 and 2 visit; ** Incidents between 2 and 3 visit; *** Incidents between 3 and 4 visit.
